# Supplementary material for: Peptide-Templated Gold Clusters as Enzyme-Like Catalyst Boost Intracellular Oxidative Pressure and Induce Tumor-Specific Cell Apoptosis
Source: Nanomaterials (Basel). 2018 Dec 12;8(12):1040. doi: 10.3390/nano8121040 (PMC6316732; doi:10.3390/nano8121040)
Supplement: Supplementary file 1 [file nanomaterials-08-01040-s001.pdf]

nanomaterials

Peptide-Templated Gold Clusters as Enzyme-like Catalyst Boost Intracellular Oxidative Pressure and Induce Tumor-Specific Cell Apoptosis

Ya Zhang, Xiangchun Zhang, Qing Yuan, Wenchao Niu, Chunyu Zhang, Jiaojiao Li, Zhesheng He, Yuhua Tang, Xiaojun Ren, Zhichao Zhang, Pengju Cai, Liang Gao *, and Xueyun Gao *

Department of Chemistry and Chemical Engineering, Beijing University of Technology, No.100, Pingleyuan, Chaoyang District, Beijing 100124, China; zhangya1@ihep.ac.cn (Y.Z.); zhangxc@ihep.ac.cn (X.Z.); yuanqing@bjut.edu.cn (Q.Y.); niuwc@ihep.ac.cn (W.N.); zhangcy@ihep.ac.cn (C.Z.); lijiaojiao@ihep.ac.cn (J.L.); hezs@ihep.ac.cn (Z.H.); tangyh@ihep.ac.cn (Y.T.); xjren@bjut.edu.cn (X.R.); zhangzc@ihep.ac.cn (Z.Z.); caipj@ihep.ac.cn (P.C.)

* Correspondence: gaoliang@bjut.edu.cn (L.G.); gaoxy@ihep.ac.cn (X.G.); Tel.: +86-10-8823-6709 (X.G.)

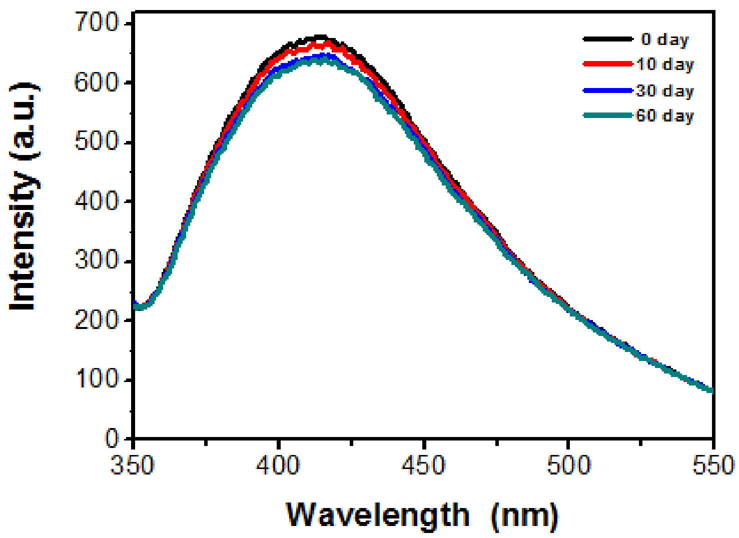

Figure S1. Stability investigation of as-prepared AuCs in 60 days by tracing the fluorescence intensity.

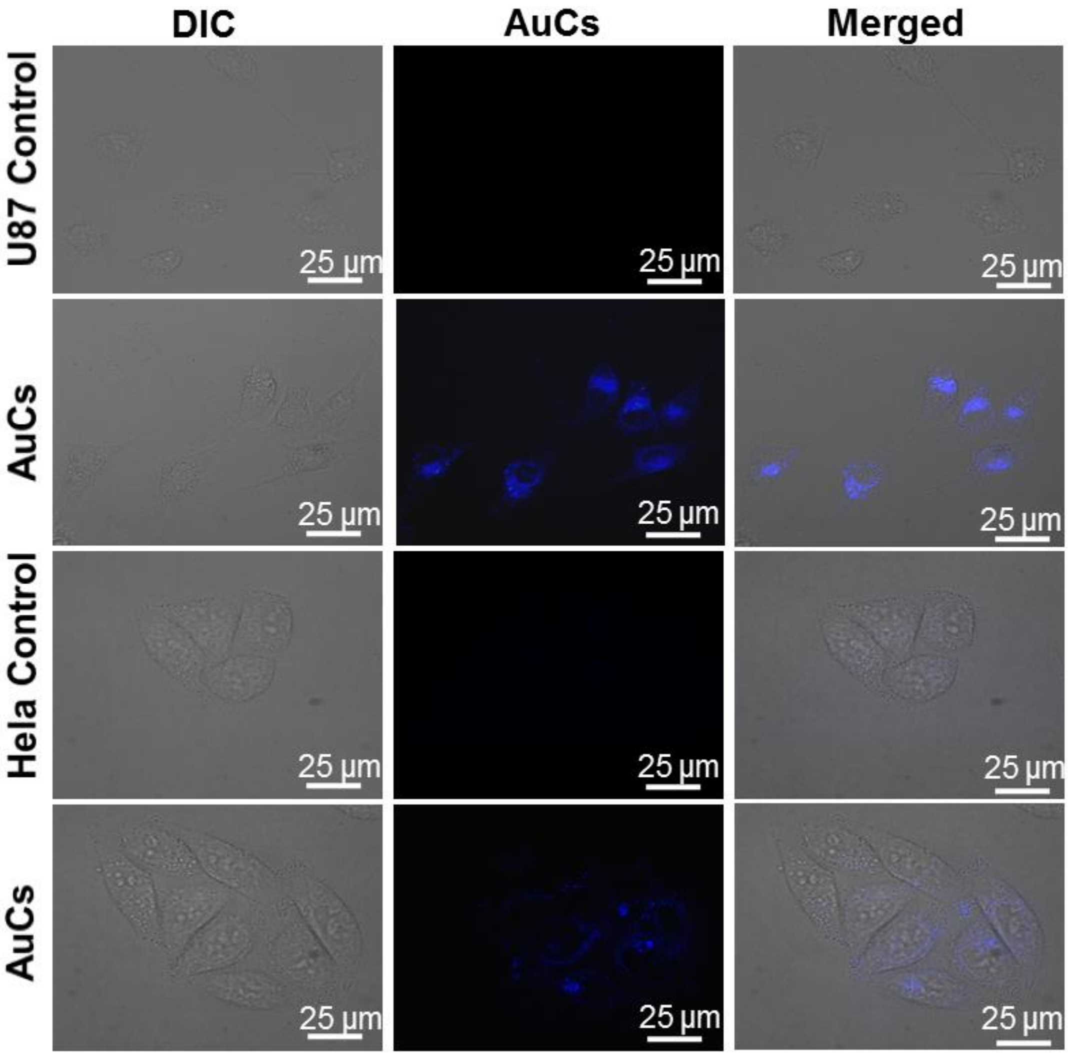

Figure S2. CLSM images of U87-MG and HeLa cells incubated with 40 μM AuCs for 24 h. The excitation wavelength is 405 nm, and blank cells were used as control.

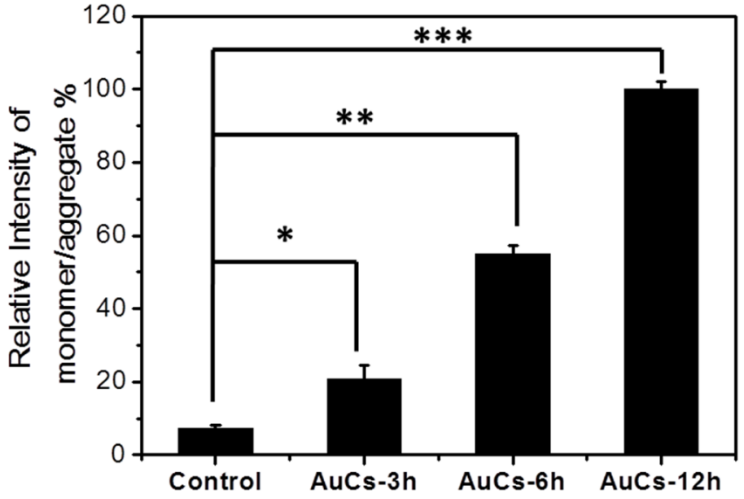

Figure S3. Statistical results of relative fluorescence intensity indicating mitochondrial membrane potential change. The ratio of green and red fluorescence intensity of cells incubated with AuCs for 12 hours was set to 100%. *** $P < 0.01$.
